# The Safe System Approach and technology—what works?

**DOI:** 10.3389/fpubh.2024.1457616

**Published:** 2024-10-09

**Authors:** Nicole Booker, Glendedora Dolce, Simon Patrick Obi

**Affiliations:** Johns Hopkins Bloomberg School of Public Health, Johns Hopkins University, Baltimore, MD, United States

**Keywords:** high-income countries (HICs), low-and lower-middle-income countries, Safe System Approach, technology, injury prevention

## Abstract

The Safe System Approach is an evidence-based strategy committed to eliminating fatalities and serious injuries among all road users. The Safe System Approach as developed in Sweden, acknowledges that human errors will occur, but the cost of these mistakes should not be death or serious injury. Technology is an integral component of the Safe System Approach and can address equity and reduce human error among other safety benefits. A literature review will be conducted to compare high-income countries leveraging the Safe System Approach and assess opportunities for technology interventions in low- and middle-income contexts. Evidence will be analyzed, as well as implementation considerations of the recent National adoption of the Safe System Approach in the United States. As SSA evolves in a global context, further evaluation is needed on the role of technology and how government policies can restrict or advance its implementation.

## Introduction

Road traffic crashes continue to affect lives across the world with traffic crashes being a leading cause of death among children and young people ages 5–29, and the 12th leading cause of death among all ages as of 2019 (1). In 2021, over 1.19 million people died from road traffic crashes with a global estimate of 15 road traffic deaths per 100,000 population (1). Though some regions across the world have continued to be disproportionately burdened by road traffic deaths, other regions have experienced progress in improving road safety (1). Formally adopted by Sweden and the Netherlands in the 1990's, the Safe System Approach (SSA) has significantly contributed to decreasing crashes in the European region and Western Pacific Region (1). The SSA is a revolutionary road safety framework that aims for zero fatalities and serious injuries (3). Adopters of the SSA have shown that preventing and reducing traffic deaths is possible. The European Region is the largest adopter of the SSA and has experienced the largest drop in road traffic deaths since 2010 at 36% (1). Furthermore, as the second largest adopter of the SSA, Western Pacific region experienced similar results with a drop in traffic deaths of 16% since 2010 (1). With technology being a notable component of SSA, this review seeks to understand how technology plays a pivotal role in crash reduction. Furthermore, with 92% of traffic deaths reported occurring in low- and middle-income countries (1), this review will examine gaps, limitations, and opportunities for technology driven by the SSA in regions that continue to be disproportionately burdened by traffic crash deaths.

## The safe system framework and technology

The SSA reinforces the idea that death and serious injuries are unacceptable (3). As an interventional framework, SSA refutes the traditional downstream theory of “human error” as a main contributing factor of road traffic injuries. Instead, SSA anticipates human errors by leveraging multi-dimensional countermeasures to achieve optimal safety for all road users (3). The foundation of SSA is based on five principles: “humans make mistakes, humans are vulnerable, responsibility is shared, safety is proactive, reducing risks is vital” (3). Based on these principles, countermeasures also known as “pillars” are defined as “safe road users, safe vehicles, safe speeds, safe roads, and post-crash care” (3). Within these pillars, technology has been known to play a pivotal role in preventing and decreasing crashes (4). According to Tingvall et al., vehicle safety technology can address SSA fundamentals such as “humans make mistakes, humans are vulnerable, and safety is proactive” (4). Technology that involves automation such as automatic braking systems and stability control, are solutions to accommodating human error (4). In-vehicular systems such as airbags, seat belts, and pedestrian detection can help to reduce crash force within human injury tolerance (4). In addition, proactive safety can be achieved by mandating technological safety standards (4).

Within Sweden, technology such as seat belt reminders, alcohol interlocks, and automatic speed cameras, have been recognized as effective in accommodating human error and contributing to improving driving behaviors on Swedish roadways (2). Research has shown that drivers were more likely to use their seat belts in vehicles with seat belt reminders than in vehicles without seat belt reminders (2). Alcohol interlocks have also played a significant role in combatting alcohol- impaired driving crashes (2). In addition, automatic speed cameras are proven to reduce speed- related crashes and injuries in SSA adopted countries (2). In Sweden, spot speed cameras are used to lower speeds and have been shown to “reduce the number of fatalities by 30% and number of people killed or seriously injured by 25%” (2). In the Netherlands, mobile speed cameras are used and have been effective in decreasing injury crashes by 21% and speed offenders from 27.4% to 15.6% on speed enforcement roads (2).

With low- and middle-income countries experiencing traffic deaths at high numbers (1), an SSA approach integrated with technology may help to combat this crisis. A review of databases and gray literature was conducted to search for SSA technology examples. Nigeria, Malaysia, and the United States were selected due to their varying economies, populations, and SSA implementation strategies to examine gaps, opportunities, and successes (Table 1).

**Table 1:** Safe System Approach (SSA) technology case studies.

**Country SES**	**Country**	**Technology**
Low-income	Nigeria	Speed Limiter Devices
		Nigeria Road Assessment Program (nRAP)
		Automated Number Plate Recognition (ANPR)
Middle-income	Malaysia	Automated Awareness Safety System (AwAS) Cameras
		Intelligent Speed Adaptation System (ISA)
		ASEAN New Car Assessment Program (ASEAN NCAP)
High-income	United States	Advanced Driver Assistance Systems (ADAS)
		Automated Enforcement (AE)

## SSA technologies across low-middle-high-income countries

Each LMIC has unique contextual factors to consider when implementing the SSA. Factors like technological development and social economic factors cause SSA interventions to vary. High income countries must consider context as well; despite the US having more resources, access, and tech advancement, they have one of the worst per capita fatality rates among developed economies (5). Below, we highlight three countries with varying income statuses that have embraced the SSA. We explore the extent of their incorporation of technology and assess the impact on their road safety outcomes.

## Malaysia

Malaysia is an upper-middle-income country with a population of 33.93 million and a Gross Domestic Product (GDP) of 407 billion dollars (6). In 2007, the Ministry of Transport Malaysia established the Malaysian Institute of Road Safety Research (MIROS) as the organization tasked with conducting research, developing objectives, and enhancing knowledge of road safety in Malaysia (7, 8). MIROS also serves as Malaysia's database for crash fatality and injury data (8). Traffic crashes in Malaysia have historically been concerning; 50% of fatalities reported were motorcyclists and at one point Malaysia was described as having the highest fatality risk in the world (9, 10). However, with strategic action taken by leaders toward road safety improvements, fatalities have been steadily declining with a rate of 22.56 per 100,000 people reported in 2016 and 18.9 per 100,000 people reported in 2019 (11). In WHO's 2023 Global Status Report, Malaysia's rate has continued to decrease to 13.9 per 100,000 people (1).

### SSA in Malaysia

In 2022, Malaysia released the 2022–2030 Malaysia Road Safety Plan, outlining 10 safety priorities that align with the United Nations' second Global Decade of Action for road safety and the Sustainable Development Goals (SDGs) 3.6 and 11.2 (11–13). This is the second national plan in the last two decades for increasing awareness of road safety and preventing injuries and fatalities (11). Priorities in the 2022–2030 plan include motorcycle safety and leveraging technology such as ABS, Automated Awareness Safety System (AwAS) cameras, and Intelligent Speed Adaptation Systems (ISAs) to reduce speeds and in turn, reduce the severity of crashes (11).

### Technology and SSA in Malaysia

1. **Automated Awareness Safety System (AwAS) cameras:** AwAS [formerly known as Automated Enforcement System (AES)] was identified in the 2006–2010 Malaysia Road Safety Plan and was implemented in 2012 (7). AwAS cameras can detect speed and red- light violations and can be strategically placed in high crash-risk locations. Evaluations have been conducted on AwAS technology in Malaysia proving it has been effective in reducing red light violations (14), increasing perception of the probability of receiving tickets, and increasing speed compliance, in accident-prone areas (15).
2. **Intelligent Speed Adaptation System (ISA):** Used by both commercial and private vehicles, ISA alerts drivers when they exceed the speed limit and can automatically limit vehicle speed. An evaluation of an ISA warning system in Malaysia was conducted in 2010 and found a significant reduction in average and maximum speed with no lasting effects once the system was removed (16). The system was preferred over an active accelerator pedal (AAP), which is an in-car speed management system designed to prevent speeding, and participants were willing to continue using the warning system after the trial (17).
3. **ASEAN New Car Assessment Program (ASEAN NCAP):** Malaysia is a member of the ASEAN NCAP, a 10-member cohort of Asian countries established in 2011 (18). In 2014, the ASEAN Transport Minister appointed MIROS as the ASEAN Road Safety Center (18). This center aims to promote and provide knowledge on road safety issues among ASEAN Member States, including road traffic laws and regulations, data management, standards development, and road safety awareness and education (18). The funding for the first phase of this program was provided by Global NCAP (18). This program contributes to the “safer vehicle” component of the SSA since all aspects of the initiative are to improve the safety of cars within the region (18).

## Nigeria

Nigeria is a lower-middle-income country (19). The Federal Road Safety Corps (FRSC) is the lead agency for road safety in Nigeria. FRSC was established in 1988 by the Federal Government of Nigeria under Decree No. 45 and is fully funded by the Federal Government of Nigeria through the National budget (20). The FRSC was judged to be one of the outstanding lead agencies within Sub-Sahara Africa responsible for road safety management (21). With a population of over 213 million, Nigeria has some 15.19 million registered vehicles on its roads. Nigeria also has a higher road fatality rate (17 deaths per 100,000 population) compared with the global average of 15 per 100,000 population (1).

### SSA in Nigeria

Nigeria, through the FRSC, has adopted the SSA through its medium-term sector strategy referred to as the National Road Safety Strategy (NRSS) 2016–2020 (22). The NRSS was designed to function according to the SSA, as emphasized in the Accra Road Safety Declaration of 2007 and highlighted in the 2010 UN Decade of Action for Road Safety recommendations (23). While the Safe System Approach is acknowledged to be part of the NRSS 2016–2020, there is a notable absence of evidence indicating a solid commitment to its implementation which raises concerns about whether the adoption of the safe system in Nigeria is supported by concrete actions.

### Technology and SSA in Nigeria

1. **Implementation of Speed Limiter Devices in Vehicles:** These devices are vehicle-specific and connected to vehicles' throttle control systems. The device prevents a driver from accelerating beyond a set speed (24). In 2016, the Nigerian FRSC adopted and enforced the use of speed limiter devices for fleet operators. These devices control the maximum speeds of equipped vehicles and serve as a tool for speed management (25). The introduction of speed limiter devices by ABC and Peace transport company resulted in significant savings in crash reduction and fuel efficiency (26). The FRSC cited the Nigerian Road Traffic Regulations ACT 2016, as amended, as the legal framework empowering the implementation of speed limiter devices.
2. **Nigeria Road Assessment Program (nRAP):** In February 2021, nRAP was established. Based on the International Road Assessment Program (iRAP), nRAP was created to improve current road safety engineering practices and establish secure road infrastructure for all users. nRAP uses mobile technology to assess roads on a five-star scale. The assessment aims to align with the Global Road Safety Performance Targets drawn from Pillar 2 of the United Nations Decade of Action for Road Safety which establishes the need for all roads to be built to a 3-star or better standard (27).
3. **Automated Number Plate Recognition (ANPR):** ANPR is a type of Automatic Enforcement System used in Nigeria. ANPR utilizes camera to capture license plate information of traffic safety violators. The government of Lagos State, a sub- national unit in Nigeria, launched the implementation of ANPR technology in 2018. In a recently published report, the ANPR captured over 850,000 traffic violations within 15 months (28). One limitation of the ANPR program in Nigeria is that it is not a national program and has only been implemented in Lagos State.

## United States

The United States (US) is a high-income country. It is one of the world's most developed countries with a population of 336 million people, a broad network of roads and highways (29), and some 275 million registered vehicles. Despite its relative wealth, the US is confronted with serious road safety challenges with 40,990 fatalities reported in 2023; this represents a 3.6% decrease from 2022, when 42,514 people were killed on US roadways (30). The National Highway Traffic Safety Administration (NHTSA) was established by the Highway Safety Act of 1970 (31). NHTSA is the lead agency for road safety in the U.S.

### SSA in the U.S.

In alignment with the SSA, the U.S. Department of Transportation has developed the National Roadway Safety Strategy (NRSS), which aims to achieve zero roadway fatalities and serious injuries (32). This strategy includes initiatives focused on infrastructure, human behavior, responsible oversight of the vehicle and transportation industry, and emergency response (32). The implementation of the NRSS is structured around the five elements of the SSA (32).

The US uses the terms Safe System and Vision Zero interchangeably, as does the Swedish practice. However, the definition of both terms can be inconsistent among jurisdictions. Some locations follow the definitions of the concept's originators, others have adopted the name but do not follow every component of the original practice (33).

### Technology and SSA in the United States

1. **Advanced Driver Assistance Systems (ADAS):** ADAS includes vehicle safety features like lane departure warning, automated emergency braking, adaptive cruise control, and blind-spot recognition (34). These systems use sensors, cameras, and radar to monitor the vehicle's surroundings and will issue a warning or intervention as needed (34). Studies have shown that ADAS systems reduce crashes significantly, with automatic emergency braking reducing rear-end crashes by 50% (35).
2. **Automated Enforcement (AE):** AE systems operate automatically via cameras that capture offender license plate information and send a citation to the registered owner (36). They have been used in the U.S. since 1987 and the three most common types of technologies include red-light cameras, speed safety cameras, and school bus stop-arm cameras (36). The permitted use of AE systems varies by state and local municipalities and in some places, approval has been rescinded after community concern surrounding citation revenue (36). The World Bank, along with several U.S. safety agencies and road safety organizations have deemed AE effective; one Insurance Institute for Highway Safety (IIHS) study found red-light cameras to reduce fatal red-light running crashes and all fatal crashes at signalized intersections by 21% and 14%, respectively (37–40). Several studies have shown similar improvements with speed safety cameras, and school bus stop arm violators (41, 42).

## Discussion

**Figure 1:**
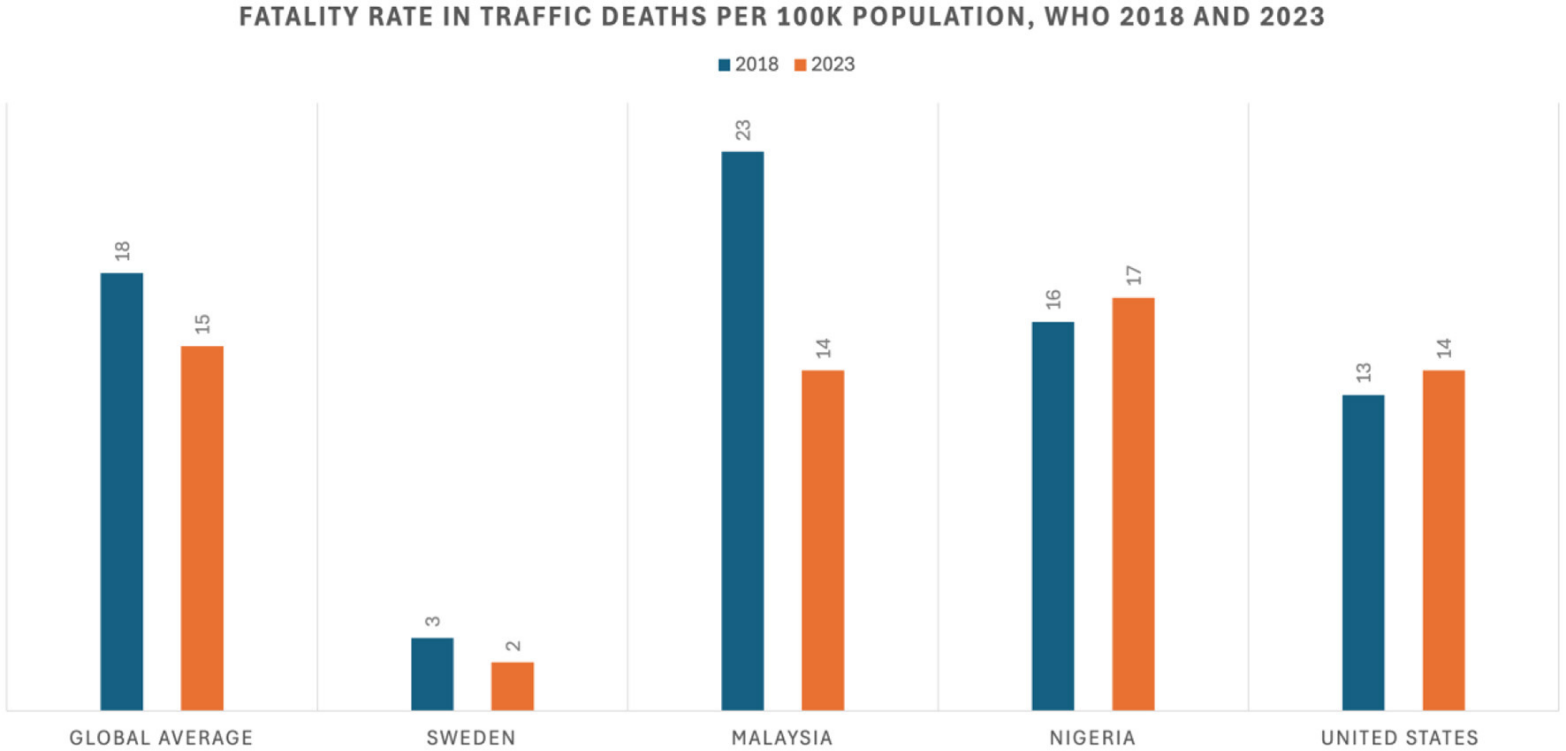
Fatality rate in traffic deaths per 100 k population, WHO 2018 and 2023 (1, 51).

After a review of these SSA countries, technology is shown to be a promising tool to combat serious traffic injuries and fatalities. However, gaps and limitations were recognized across the different country incomes that need to be addressed to effectively intervene in this ongoing crisis. Leaders in SSA adoption, such as Sweden and other Scandinavian countries tend to be higher-income nations (2). Income can be considered an advantage when leveraging technology as a strategy to decrease and prevent serious traffic injuries and fatalities. The United States shows that despite being a high-income country with resources and road safety innovation opportunities, obstacles can exist for safety technologies. The United States has a higher fatality rate than most nations of similar economic levels (5) (Figure 1); while many factors affect traffic fatality rates, the US has lower rates of adoption of certain technologies, such as automated enforcement cameras than other high-income countries (36). Separation of authorities may also be a possible factor in the low adoption of certain SSA technologies in the US. States have legislative powers which allow them to make their traffic safety laws and regulations (43); State DOT's can decide what strategies they are willing to fund and implement, including safety technology (43). Therefore, there are variations in the adoption of traffic safety approaches across 50 states. In addition, local DOT's may not have authority unless granted by the State (43). Therefore, local transportation agencies are usually left to comply with decisions made by State DOTs and face restrictions in funding (44).

While the advantages of high-income nations are clear, this does not preclude lower-income nations from adopting SSA principles. Many levels of technology can be implemented, including low-cost, quick-build solutions that are consistent with SSA principles such as red-light enforcement devices, flashing beacons at stop-controlled intersections etc. (45). Nigeria and Malaysia are examples of LMIC SSA countries that have found ways to implement technology despite GDP level and other challenges. For instance, Malaysian government officials identified AwAS cameras in their 2006–2010 Road Safety Plan, but it was not implemented until 2012; this delay could have been caused by institutional fragmentation and over dependency on limited government funding, which have been identified as challenges in road safety management in Malaysia (46, 47). In Nigeria, Uzondu et al. (48) identified inadequate funding for road safety as a critical challenge, suggesting that it contributes to the country's “overarching difficult” road safety environment (48). This finding aligns with Bishai et al. (49) who demonstrated a negative impact of insufficient road safety funding on overall outcomes (49). Differences in these government structures point out that while the SSA principles remain the same, strategies for the implementation of technology may need to be tailored according to the country's context (e.g., funding, government structure, etc.,). SSA model policy could aid in this implementation and it is recommended that it be explored.

It can be difficult to independently assess the association between technology and safety outcomes in SSA adopted countries however, according to a report by the Johns Hopkins Bloomberg School of Public Health, the Institute of Transportation Engineers, and the FIA Foundation, several nations have experienced significant reductions in traffic fatalities following the implementation of SSA (50). Implementing SSA in LMIC's can reduce road deaths and injuries, this review emphasizes the potential leveraging technology within SSA. Moreover, the affordability of SSA technologies is an important factor for LMICs to consider when designing their SSA interventions. LMICs may prioritize spending on lower-cost SSA technologies as highlighted earlier, while high-income countries may prioritize spending more resources on advanced vehicle technologies (Table 2).

**Table 2:** Safe System Approach (SSA) technology case studies.

**Country SES**	**Country**	**Technology**	**Cost**	**In country efficacy**
			**High/medium/low**	**No/low/empirical**
Low-income	Nigeria	Speed Limiter Devices	Medium	No
		Nigeria Road Assessment Program (nRAP)	Medium	No
		Automated Number Plate Recognition (ANPR)	Medium	No
Middle-income	Malaysia	Automated Awareness Safety System (AwAS) Cameras	High	Empirical
		Intelligent Speed Adaptation System (ISA)	High	Empirical
		ASEAN New Car Assessment Program (ASEAN NCAP)	Medium	Low
High-income	United States	Advanced Driver Assistance Systems (ADAS)	High	Empirical
		Automated Enforcement (AE)	High	Empirical

## Conclusion

Technology is not the only tool to achieving the Safe System Approach; however, this review found that the potential for safety advancement while leveraging technology exists across socio-economic levels. A factor identified for SSA technology implementation may be differences in leadership structure and funding commitment. There were limitations in the certainty of these conclusions, including a lack of impact analysis, and high-quality evidence on the effectiveness of technology especially in SSA adopted LMICs. As SSA evolves in a global context, further evaluation is needed on the role of technology and how government policies can restrict or advance its implementation.
